# Burnout among diabetes specialist registrars across the United Kingdom in the post-pandemic era

**DOI:** 10.3389/fmed.2024.1367103

**Published:** 2024-03-26

**Authors:** Adnan Agha, Ansu Basu, Eram Anwar, Wasim Hanif

**Affiliations:** ^1^Department of Internal Medicine, College of Medicine and Health Sciences, United Arab Emirates University, Al Ain, United Arab Emirates; ^2^Department of Diabetes and Endocrinology, Sandwell and West Birmingham Hospitals NHS Trust, City Hospital, Birmingham, United Kingdom; ^3^Department of Diabetes, Endocrinology and Lipid Metabolism, City Hospital, Birmingham, United Kingdom; ^4^Honorary Associate Clinical Professor, University of Birmingham, Birmingham, United Kingdom; ^5^NHS West Midlands, Birmingham, United Kingdom; ^6^Department of Diabetes and Endocrinology, University Hospitals Birmingham, Birmingham, United Kingdom

**Keywords:** diabetes and endocrinology specialty trainee registrar, burnout – professional, United Kingdom, bullying, work-related stress

## Abstract

**Introduction:**

Burnout syndrome is a condition resulting from chronic work-related stress exposure and can be identified by the presence of one or more of the three classic dimensions of burnout, i.e., emotional exhaustion, depersonalization, and lack of personal accomplishment, which negatively impact physician health and productivity.

**Objective:**

This study aimed to identify burnout among Diabetes and Endocrinology Specialty Training Registrars (DStRs) across the United Kingdom.

**Design/setting:**

It was a Cross-sectional observational study after ethical approval ERSC_2022_1166, utilizing the gold standard Maslach Burnout Inventory to measure burnout syndrome, and to determine self-reported stressors and compare them with the results of our previous survey in 2018.

**Participants:**

Over 430 DStRs across the United Kingdom were invited electronically through their deanery representatives and specialty training bodies.

**Results:**

Using Google Forms™ to gather data, we were able to collect 104 completed surveys. Results revealed that 62.5% (*n* = 65) of participants have burnout (5% increase from the previous survey in 2018), 38.6% (*n* = 40) have high emotional exhaustion, and 44.2% (*n* = 46) feel a lack of personal accomplishment. “General Internal Medicine specific workload” was the most common self-reported stressor reported by 87.5% (*n* = 91) of participants, whereas bullying/harassment and discrimination at work were reported by 35.6% (*n* = 37) and 30.77% (*n* = 32) of participants, respectively. Using multivariable logistic regression model, personal stress (OR, 4.00; 95% CI, 1.48–10.86; *p* = 0.006) had significant, while Bullying/harassment (OR, 3.75; 95% CI, 0.93–15.12; *p* = 0.063) had marginal impact on the presence of burnout.

**Conclusion:**

Diabetes and Endocrinology Specialty Training Registrars frequently experience burnout syndrome, which has increased over the last 4 years. However, organizational changes can help identify, prevent, and treat physician burnout.

**Trial registration:**

NCT05481021 available at https://ichgcp.net/clinical-trials-registry/NCT05481021.

## Introduction

The term “burnout” was first used in the late 1960s and was later defined by Freudenberger in 1974 as let-down or fatigue among healthcare staff due of increased demands on their physical strength and mental energy as well as a lack of resources (1). Recently, nearly half a century later, a review of approximately 200 studies has reported up to 142 definitions for physician burnout (2). The most universally accepted definition and a validated tool to diagnose burnout was introduced in 1981 by Maslach et al. (3) in the form of the Maslach Burnout Inventory (MBI). Maslach et al. defined burnout syndrome as a health condition that develops after chronic exposure to work-related stress and is diagnosed using MBI by exhibiting one or more of the three characteristic dimensions of burnout: emotional exhaustion, depersonalization, and lack of personal accomplishment (4, 5). In simpler terms, among physicians, “emotional exhaustion” (EE) equates to the feeling of running out of any emotions while dealing with patients in an emotional context by the end of the workday. Conversely, “depersonalization” (DP) refers to treating patients as objects, medical condition, or diseases rather than considering them as individuals, which results in the development of a callous or unsympathetic attitude toward them. Finally, lack of personal accomplishment (PA) relates to feeling ineffective or undervalued in terms of helping patients in achieving care or attaining professional or career achievements. Notably, burnout exists as a distinct entity that may not be related to other work stressors, depression, job dissatisfaction, and fatigue; thus, it can exist independently of other occupational conditions (5, 6).

The impact of physician burnout is widespread in healthcare and can be classified into categories related to patient care, burden on healthcare staff, and health systems infrastructure or economics-linked effects. From the perspective of patient care, adverse patient safety incidents and medical errors, including major errors, are twice as likely to occur if the staff are experiencing burnout, leading to suboptimal quality of care and decreased patient satisfaction (7, 8). Cross-sectional studies have shown poorer patient care standards and longer recovery times for hospitalized patients associated with the presence of burnout (9, 10). Burnout seems to directly affect the physical health of healthcare staff and physicians as demonstrated in a recent meta-analysis, which showed that burnout was associated with an increased risk of depression; meanwhile, another study found increased abnormal glucose metabolism in physicians with symptoms of burnout (11, 12). Burnout has also been associated with a twofold increase in employment disability and significantly increased risk of coronary events in healthcare staff (13, 14). Studies conducted in North America revealed that burnout can reduce physician job satisfaction and clinical output, which can double the self-reported intent to leave the current job (15, 16). A recent study extrapolating the single-item MBI-based measure indicated that a one-point increase in EE was associated with nearly a one-fourth reduction in professional effort and almost two-thirds decrease in work hours over the next year (17). Physician burnout can also impact their health by putting them at increased risk of substance abuse or dependence and can double their risk of suicidal ideation (18, 19). The effect of physician burnout in the healthcare system is momentous in terms of losses on the health economy, with increased physician turnover and resultant loss of productivity leading to shortfall in physicians; the healthcare system in the United States is expected to reach a national deficit of 90,000 physicians by the next 2 years (20, 21).

The prevalence of burnout in physicians is roughly twice that in the general population, which may be related to physicians’ role in dealing with patients’ lives and challenging conditions and pressures of difficult decision-making, increased workload, and ever-increasing productivity expectations (22). A recent large study in the United Kingdom (UK) showed that in the National Health Service, general practitioners have a high level of burnout among half of the physician workforce (23). Burnout is not separately measured in the UK workforce statistics database and is not distinguished from work-related stress. However, the estimated cost of workdays lost due to sickness related to burnout/work-related stress is £2.4 billion in the United Kingdom (24); in the United States, this cost is almost $5 billion (21). The authors of this paper have previously presented the results of a national survey assessing burnout among Diabetes and Endocrinology Specialty Training Registrars (D&E-StRs) across England using the MBI; the results revealed burnout in 57.5% of the D&E-StRs during the pre-COVID-19 era, with increased general internal medicine workload and lack of specialty training being the most common self-reported stressors (25). Since then, the Internal Medicine Training program has increased the number of years spent in Internal Medicine to 3 years (from 2 years) (26). After the COVID-19 pandemic, which impacted work, training, and educational activities of D&E-StRs, the authors attempted to repeat the national survey to assess the current well-being of D&E-StRs and presence of burnout syndrome. This study aimed to assess burnout in D&E-StRs in the entire UK and determine changes from the previous survey conducted nearly 4 years ago.

## Objectives

### Primary objective

The primary objective was to identify burnout syndrome among D&E-StRs across England, Wales, Scotland, and Northern Ireland using the recognized gold standard tool, i.e., MBI, under license from Mind Garden™.

### Secondary objectives

1. Identify possible self-reported stressors associated with the presence of burnout in our target population.
2. Assess any self-reported bullying/harassment or discrimination experienced during training.
3. To compare the frequency of burnout among the target population after 4 years, using the new Maslach Burnout Inventory Manual, 4th edition (4).
4. Perform multivariate analysis and modeling to identify factors that best predict burnout in our population.

## Participants and methods

### Study design and oversight

This prospective cross-sectional observational study was conducted using an online survey. Ethical approval was obtained from the United Arab Emirates University Social Sciences Ethics Committee (approval letter ERSC_2022_1166) and registered on ClinicalTrials.gov with ID number NCT05481021 as the study was conducted jointly between the authors in the UAE and UK. We sent the survey questionnaire sent to all the D&E-StRs across England, Scotland, Wales, and Northern Ireland (estimated to be 430 at the start of the study in 2022) and various deanery trainee representative helped collect data from these separate regions over a period of 1 year (2022–2023).

### Data collection

The survey questionnaire, which included demographic data, questions on discrimination, bullying/harassment, and stress-related factors, was distributed via email to D&E-StRs after obtaining informed consent. The demographic data questions included naming the current deanery/region of work, job title/position, e.g., clinical fellow, specialist traininee year 3 (ST3) etc., working full time or less than full time, age, sex, ethnicity, family details including having young kids or other family members to look after. Questions relevant to training included, “How many months in total of reduced general internal medicine (GIM) commitments do you get in your deanery during your regular training programme as dual CCT accreditation for GIM/Diabetes & Endocrinology to concentrate on your sub-specialty, i.e., Diabetes & Endocrinology,” and “How many months in total of reduced GIM commitments do you think that you should get from your deanery during your regular training programme as dual CCT accreditation for GIM/Diabetes & Endocrinology to concentrate on your sub-specialty, i.e., Diabetes & Endocrinology.” Both these were derived from our original burnout in similar population study 4 years ago (25).

Regarding the stressors, the respondents were given an open free text to describe various stressors which were sub-categorized into Workplace Specific Stressors (e.g., rotation allocation/ deanery related or hospital related issues/ bullying or discrimination etc), Diabetes & Endocrine Specialty Specific Stressors (e.g., less exposure to specialty clinics/ no rotation to hospitals with super-specialty clinics/ problems around specialty workload / problems attending conference or research etc), GIM Specific Stressors (e.g., exposure to GIM clinics/ GIM Oncalls/ problems around GIM workload etc), Personal Stressors (e.g., family issues/ visa/ financial etc), and/or Other Stressors that do not fit in the above categories.

They were also asked specific questions around experiencing or witnessing by direct observation any bullying/harassment and/or discrimination is last 2 years and if they responded “yes” the participants would provided further free text details.

### Study population and data handling

We disseminated these questionnaires with support from the Society for Endocrinology and Young Diabetologists and Endocrinologists Forum. Using Google Forms™, we collected responses between April 2022 and March 2023 to allow for maximum participation. We planned to include at least 105 participants based on sample size calculation with an estimated total population of 430 D&E-StRs with 95% confidence level and 5% margin of error, considering a minimum online response rate of 10%. All D&E-StRs with at least 3 months in post were invited to participate in the study. Details of the posts included in the survey and the deaneries represented are provided in the Supplementary files. This study excluded participants who self-reported the presence of active psychiatric illness or were under treatment and those who were out of post for more than 3 months (e.g., on sick leave). The respondents were given a time frame of 3 months to respond to the survey and then reminded at 3 monthly intervals and data collection was completed at 12 months. It was a national study across United Kingdom with all data collected from various deaneries collated at lead site at Burton-on-Trent and deaneries names were anonymized by allocating them numbers and then the data analysis was performed at a different site in Birmingham.

We exported the survey responses from Google Forms™ into an MS Excel spreadsheet, securely stored on the UAE university computer with access limited to the principal investigator (AA). No personal identifiable information was collected or stored, and data were strictly anonymized prior to analysis. The self-reporting stress-related factors included in the questionnaire were similar to those in our previous burnout survey (25) and were grouped under workplace/deanery-related, specialty-related (Diabetes and Endocrinology [D&E]), related to GIM, and personal factors that may contribute to stress; a separate free-text section was also available to the participants. We used binary questions to collect information on bullying/harassment. The full list of the questions used in the survey is presented in the Supplementary files.

### Data analyses

The primary outcome of interest was trainee burnout. We used the Maslach Burnout Inventory, 4th edition (2016), in our questionnaire after obtaining license from Mind Garden™ (4). We defined the presence of high burnout as reaching the cutoff value in either of the three dimensions based on scoring the responses on the three subscales of burnout, i.e., EE, DP, and lack of PA. Further, we defined the cutoff score for each dimension as high burnout if the participant’s score reached standardized z-value, with EE cutoff value calculated as mean + (standard deviation [SD] × 0.5) (range, 0–54), DP cutoff value as mean + (SD × 1.25) (range, 0–30), and lack of PA cutoff value as mean + (SD × 0.10) (range, 0–48) according to the recommendations of Maslach et al. (4). For EE and DP, higher scores indicated worse outcomes, whereas for PA, higher scores indicated a better outcome. Continuous variables are presented as means and SD or as medians with interquartile range as appropriate. Categorical variables are expressed as frequencies and percentages. Multiple imputation of missing data was not performed as no missing responses were encountered. A multivariable logistic regression was undertaken for the primary analysis to identify the effect of demographic factors, stress-related factors, bullying/harassment, and discrimination on trainee burnout in order to understand the main effects and not to develop a predictive model.

All statistical analyses were undertaken using STATA/MP version 16.1 (StataCorp). All *p*-values are two-sided, and an α-value of 0.05 was considered to indicate statistical significance.

## Results

A total of 104 D&E-StRs from all the Health Education England deaneries in England, Scotland, Wales, and Northern Ireland completed this survey; response rate of 24.1% with an estimated total of 430 DStRs. Most participants were male (57; 54.9%); one participant preferred not to declare their sex. The latter individual was not included in the multivariate analyses as sex was used as a covariate; all other analyses used the entire cohort. All responders were included in the demographic statistics (Table 1). Most trainees (85; 81.7%) were in the age group of 31–40 years. A nearly equal proportion of the trainees across the region identified themselves as Asian/Asian–British (47.1%) or British/European (41.4%). Nearly a third (57.7%) of the trainees were single (family size = 0); these were mostly in the 31–35-year age group. As expected, results revealed a strong correlation between family size and age group with younger students with smaller families (*r*^2^ = 0.53; *p* < 0.0001). More than three-fourths of the trainees had full-time employment (83/104; 79.8).

**Table 1:** Demographic characteristics of the participants.

Characteristics	No. (%); *N* = 104
Age group (in years)	
<31	8 (7.7)
31–35	60 (57.7)
36–40	25 (24.0)
41–45	9 (8.7)
>45	2 (1.9)
Female Gender	46 (44.2)
Ethnicity	
Asian/Asian-British	49 (47.1)
British/European	43 (41.4)
Afro-Caribbean	1 (1.0)
Arab	3 (2.9)
Others	4 (3.9)
Prefer not to say	4 (3.9)
Family size (kids/dependants)	
0	39 (37.5)
1–2	45 (43.3)
> = 3	20 (19.2)
Posts held	
Clinical fellow/non-training post	63 (5.8)
Academic trainee / Academic Clinical fellow	3 (2.9)
Internal Medicine trainee year 2/3 awaiting training number	1 (1.0)
Out of programme trainee	2 (1.9)
Specialist trainee year 4	17 (16.4)
Specialist trainee year 5	25 (24.0)
Specialist trainee year 6	30 (28.9)
Specialist trainee year 7	13 (12.5)
Specialist trainee year 8/ new consultant within 3 years of certificate of completion of training	4 (3.9)
Clinical Lecturer	3 (2.9)

GIM training commitments varied widely across the deaneries, with some deaneries offering more than 24 months of GIM training-free period for D&E-StRs (8/104; 7.7%), possibly to allow more focus on D&E specialist training; in contrast, other deaneries had 67 (64.4%) participants with no GIM-free training period included in their program. This variation remains unchanged from our earlier survey before the COVID-19 pandemic in 2018 (25, 27) As expected, 88% (59/67) of those who had no GIM-free training periods opted for fewer GIM commitments in the survey. Overall, 76 trainees (73.8%) would have liked to do undertake fewer GIM commitments to allow more D&E specialty exposure during their training.

Among the trainees, GIM-specific work, including out-of-hours work, appeared to be the most common factor that induced stress (91/104 [87.5%]) among the four main categories of stress factors self-reported in the survey (Table 2). Only four (3.9%) trainees in the survey did not experience any form of stress. Among the four documented categories of stress factors, only personal stress appeared to have a significant impact on the final outcome of burnout (OR, 3.51; 95% CI, 1.38–8.99; χ^2^ = 8.68; *p* = 0.0032) in the univariate analysis. This remained significant even after adjusting for all other stress-related factors (OR, 3.63; 95% CI, 1.46–9.05; *p* = 0.006).

**Table 2:** Self-reported stress factors by participants.

	stress factors reported by participants	Participants (%); *N* = 104
1	Stress related to diabetes and endocrinology training	74 (71.2)
2	Stress related to General Internal Medicine training	91 (87.5)
3	Personal stress affecting training	69 (66.4)
4	Any other work-related stress	82 (78.9)
Total participants reporting stressors		100 (96.2)
Participants reporting no stressors		4 (3.8)

Bullying/harassment was reported by 37 (35.6%) trainees, while 32 (30.77%) reported experiencing discrimination at work. Additionally, results showed no association between the trainee’s sex and experience of bullying/harassment (*r*^2^ = 0.04; *p* = 0.72) or discrimination (*r*^2^ = 0.05; *p* = 0.62) on multivariate logistic regression. Similarly, these two factors were not influenced by the ethnic background of the individual trainee (for harassment *r*^2^ = 0.10; *p* = 0.31 and for discrimination *r*^2^ = −0.07; *p* = 0.47).

The cutoff value identified for the burnout dimension for D&E-StRs was >27 for EE, >17 for DP, and < 12 for PA, as per Maslach burnout inventory standardized formula (4). Using this criterion, 62.5% (*n* = 65) of the participants were found to have high burnout in at least one of the dimensions. Among the 65 participants with high burnout, 44.6% (*n* = 29) had high burnout in more than one dimension (16 participants in two and 13 in all three dimensions). Furthermore, lack of PA was the most frequent burnout dimension present among the trainees (46/104; 44.2%), followed by EE (40/104; 38.6%) and DP (21/104; 20.2%).

We used a multivariable logistic regression model to assess the impact of independent predictors on the primary outcome of trainee burnout (see Table 3). When adjusted for other covariates, only personal stress appeared to have a significant impact on burnout (OR, 4.00; 95% CI, 1.48–10.86; *p* = 0.006). Other factors inducing stress—such as work (OR, 0.99; 95% CI, 0.30–3.28; *p* = 0.995), GIM training (OR, 0.77; 95% CI, 0.19–3.08; *p* = 0.711), or D&E training (OR, 0.98; 95% CI, 0.33–2.85; *p* = 0.996)—did not have any impact on the primary outcome. Bullying/harassment (OR, 3.75; 95% CI, 0.93–15.12; *p* = 0.063) but not discrimination (OR, 0.70; 95% CI, 0.17–2.94; *p* = 0.629) may have had marginal impact on burnout, but this impact was not statistically significant. Sex, race, or age group did not have any impact when adjusted for other variables.

**Table 3:** Multivariable logistic regression of the variables.

Variable	Odds-ratio	95% CI	*p*-value
Gender			
Male	1.00		
Female	1.84	0.72–4.73	0.203
Race*			
Asian	1.00		
Caucasian	0.77	0.27–2.20	0.629
Other groups	3.69	0.36–38.09	0.273
Age category**			
<31 years	1.00		
31–35 years	0.82	0.12–5.54	0.835
36–40 years	0.36	0.05–2.82	0.333
41–45 years	0.29	0.03–3.17	0.307
Self-reported stressors			
Work-related stress present	0.99	0.30–3.28	0.995
Diabetes & Endocrinology-related stress	0.98	0.33–3.85	0.996
G(I)M-related stress	0.77	0.19–3.08	0.711
Personal stress	4.01	1.48–10.86	0.006
Bullying/harassment and discrimination			
Discrimination	0.70	0.17–2.94	0.629
Harassment	3.74	0.93–15.12	0.063

*Only 1 Afro-Caribbean trainee which was dropped by the model (OR = 1.00).

**Only 2 trainees > 45 years and hence dropped by the model (OR = 1.00).

The details of the bullying/harassment and discrimination provided by six participants only included comments like “Some senior nurses/ward managers were very difficult to work with,” “Toxic people with unsafe and dangerous working environment,” “Bullying and scapegoating was the norm,” “Pressurizing and harassment by medical staffing and medical director to do on-call when not scheduled to do on-calls,” “Hospital bullying and victimization,” and “Consultant belittled him, made him feel very incompetent.” Similarly, for those experiencing discrimination, only seven respondents clarified with further comments: “I sometimes feel left out,” “Very discriminatory attitude from some consultants and made to feel very low and extremely depressed,” “I felt my health/life was at risk in that rotation,” “Mainly from patients on telemedicine appointments (prejudice against foreign last name),” “One of my colleagues was shamed in front of other team members,” “Experienced bullying on escalating the deficiencies in training opportunities,” and “Our colleagues (African ethnic origin and South-Asian origin) were twice verbally abused by different consultants (local/white ethnicity), which left a really bad impression.”

We performed an exploratory analysis using personal stress as the outcome variable with sex, race, age group, and family size as covariates to ascertain whether these have any predictive value. In this model, personal stress was greater with increasing family size: no children (OR, 1), 1–2 children (OR, 5.64; 95% CI, 1.87–17.01; *p* = 0.002), and more than 3 children (OR, 9.49; 95% CI, 1.89–47.52; *p* = 0.006). However, it had no bearing on the individual’s sex or race.

## Discussion

This is the second burnout survey since the first survey completed prior to COVID-19 in 2018 (25). This survey included Northern Ireland deanery (not included in the first survey), but the participants in both surveys, although 4 years apart, were similar in sample size—106 participants in first survey and 104 in current one (25). Burnout syndrome was identified in 57.5% (61/106) of participants in 2018; this number has now increased to 62.5% (65/104) of participants. This denotes an increase of 5% over 5 years. The high EE remains relevant (38.6%; 40/104) in the current survey (in comparison with 45.2% or 48/106 in 2018), but the lack of PA has increased from 24.5% (26/106) to 44.2% (46/104), which makes it the most frequent dimension of burnout present among D&E-StR in the survey. Among the self-reported stress-related factor, “GIM-specific workload” is the most common factor, which has increased from 60.4% (64/106) in 2018 to 87.5% (91/104) in 2023, with only four (3.9%) trainees currently reporting an absence of stress at workplace. This survey additionally included bullying/harassment (35.6%; 37/104) and experiencing discrimination at work (30.77%; 32/104), which was not previously included. Although we reported a lot of examples of bullying, harassment and discrimination experienced by the participants in this survey we did not specifically measure this objectively.

To the best of our knowledge, there has no other published research on burnout among this specific target population, however burnout has been reported in doctors working in UK as high as 51.2% in domestic medical graduate and slightly lower 42.9% in international medical graduates, using the data from optional section of the national trainee survey, (28) but this was based on Copenhagen burnout inventory, a much less validated and reported tool in literature as compared MBI. Our study showed an even higher frequency of 62.5% in doctors who are at higher level of training (in specialty training in D&E). Another recent study evaluating the burnout in trainee doctors in UK found that lower socioeconomic status can worsen the likelihood of burnout, and the burnout was highest among internal medicine trainees (29). Similarly, we found that our participants with personal stressors (including socioeconomic factors) have a significantly increased risk of experiencing burnout (OR 4.00; *p* = 0.006).

Nearly one-third of the participants in our study reported bullying and feeling discriminated, and although there no studies specifically looking into this phenomenon particularly among our target population, there is recent systematic review of studies involving nursing students which reported workplace bullying as high as 58.2% and discrimination in the form of racism being 12.2% (30). Another study in Australia, identified uncivil and unprofessional behaviors experience by all medical staff including doctors, nurses, paramedical and support staff at workplace to be high which impacted on their wellbeing (31). Our study showed that bullying/harassment had negative impact on participants but this association did not reach statistical significance (*p* = 0.063) while experiencing discrimination did not have any impact on burnout.

Studies on physician burnout have described factors that induce and/or trigger stress. However, literature on how to tackle burnout among vulnerable individuals remains scarce. In line with the recommendations of the United States National Academy of Medicine consensus report, (32) it is essential to adopt a systematic approach focusing on improving the working environment rather than just providing resilience training to physicians in order to achieve large-scale organizational changes. Similar multi-pronged approach, addressing the mental health conditions such as burnout among physicians in UK has been suggested, including but limited to promoting friendly and supportive work environment, and establishing open dialogue practices to bring forward any occupational stressors as well as structural organizational transformations to support mental health and address burnout syndrome (33).

### Strength and limitations

This is the second national study by authors on D&E-StRs across all deaneries in United Kingdom which assesses burnout and possible stressors among the participants. It also indicates an increase in burnout among the respondents since the first study 5 years ago. Addressing the core stressors and reducing burnout can improve the work performance and physician satisfaction in D&E-StRs.

Our study was limited by its online and remote nature, thereby reducing the response rate to 24.1%. Further, inferences were limited owing to the observational design of the study without screening for pre-existing stress or mental health problems, which run the risk of selection bias. We did not use any scale to measure bullying and discrimination and our survey did not ask any specific questions about reporting such incidents to the administration. We also did not measure physician resilience in this survey, which may be a helpful tool for planning intervention strategies among physicians experiencing burnout.

## Conclusion

Physician burnout is a serious condition that may have long-term effects on physician health, mental well-being, and productivity and may lead to decreased organizational efficiency. In this study assessing burnout syndrome among D&E StRs across UK, conducted nearly 4 years after the authors’ previous pre-pandemic work, we were able to identify burnout syndrome in 62.5% of the participants which is an increase by 5% and it highlights the need for further research on burnout syndrome and planning of organizational strategies to prevent and treat physician burnout. While direct comparison may not be possible as the respondents of the two surveys were not the same, we inferred that the problem of burnout may be more widespread with lesser impact related to the COVID-19 pandemic. In our study, we were able to highlight and describe the various stressors that may impact burnout. We also discovered that one-third of our participating D&E StRs reported experiences of bullying and/or discrimination, which warrants further research into this subject.

## Data availability statement

The raw data supporting the conclusions of this article will be made available by the authors, without undue reservation.

## Ethics statement

The studies involving humans were approved by United Arab Emirates University Social Sciences Ethics Committee (approval letter ERSC_2022_1166). The studies were conducted in accordance with the local legislation and institutional requirements. The participants provided their written informed consent to participate in this study.

## Author contributions

AA: Conceptualization, Data curation, Formal analysis, Investigation, Methodology, Project administration, Resources, Supervision, Validation, Visualization, Writing – original draft, Writing – review & editing. AB: Formal analysis, Methodology, Software, Validation, Writing – review & editing. EA: Conceptualization, Data curation, Methodology, Resources, Visualization, Writing – review & editing. WH: Funding acquisition, Resources, Validation, Writing – review & editing.
