# Supplementary material for: Burnout among diabetes specialist registrars across the United Kingdom in the post-pandemic era
Source: Front Med (Lausanne). 2024 Mar 26;11:1367103. doi: 10.3389/fmed.2024.1367103 (PMC11003518; doi:10.3389/fmed.2024.1367103)
Supplement: Supplementary file 1 [file Data_Sheet_1.docx]

**Supplementary file 1: Details of participants of the survey**

The participants of this survey included Diabetes and Endocrinology Specialty trainee Registrars (D&E-StRs) from all deaneries across England, Scotland, Wales and Northern Ireland including:

o Health Education Yorkshire & Humber

o Health Education East Midlands

o Health Education East of England

o Health Education Kent Surrey & Sussex

o Health Education North West & Mersey Deanery

o Health Education North East

o Health Education South West Peninsula and Severn Deanery

o Health Education Thames Valley (Oxford)

o Health Education Wessex

o Health Education West Midlands

o London Deanery (North West, North Central & East London, South London)

o Northern Ireland Medical & Dental Training Agency

o Scotland Deanery (West, South East, East, North)

o Wales Deanery associated with the following deaneries in United Kingdom

The Diabetes and Endocrinology Specialty trainee Registrars (D&E-StRs) participants were holding the position of Specialist trainee in Diabetes and Endocrine with national training number from year 4 to year 8 but also included trainees without national training number in the following post

- Clinical fellow/ non-deanery training post/locally employed specialty trainee doctor

o Academic clinical fellow trainee (ACF)

o Core Trainee or Specialist trainee year 3/ awaiting National Training Number

o Associate Specialist Equivalent/Trust middle grade

o Clinical Lecturer

o Diabetes consultant (new i.e within 3 years of appointment, locum, fixed term or substantive)
