# Supplementary material for: Burnout among diabetes specialist registrars across the United Kingdom in the post-pandemic era
Source: Front Med (Lausanne). 2024 Mar 26;11:1367103. doi: 10.3389/fmed.2024.1367103 (PMC11003518; doi:10.3389/fmed.2024.1367103)
Supplement: Supplementary file 2 [file Data_Sheet_2.pdf]

Supplementary file 2: Details of the participants of the survey

The participants of this survey included Diabetes and Endocrinology Specialty trainee Registrars (D&E-StRs) from all deaneries across England, Scotland, Wales and Northern Ireland including:

- Health Education Yorkshire & Humber
- Health Education East Midlands
- Health Education East of England
- Health Education Kent Surrey & Sussex
- Health Education North West & Mersey Deanery
- Health Education North East
- Health Education South West Peninsula and Severn Deanery
- Health Education Thames Valley (Oxford)
- Health Education Wessex
- Health Education West Midlands
- London Deanery (North West, North Central & East London, South London)
- Northern Ireland Medical & Dental Training Agency
- Scotland Deanery (West, South East, East, North)
- Wales Deanery associated with the following deaneries in United Kingdom

The Diabetes and Endocrinology Specialty trainee Registrars (D&E-StRs) participants were holding the position of Specialist trainee in Diabetes and Endocrine with national training number from year 4 to year 8 but also included trainees without national training number in the following post

- Clinical fellow/ non-deanery training post/locally employed specialty trainee doctor
- Academic clinical fellow trainee (ACF)
- Core Trainee or Specialist trainee year 3/ awaiting National Training Number
- Associate Specialist Equivalent/Trust middle grade

- Clinical Lecturer
- Diabetes consultant (new i.e within 3 years of appointment, locum, fixed term or substantive)
