# Supplementary material for: Burnout among diabetes specialist registrars across the United Kingdom in the post-pandemic era
Source: Front Med (Lausanne). 2024 Mar 26;11:1367103. doi: 10.3389/fmed.2024.1367103 (PMC11003518; doi:10.3389/fmed.2024.1367103)
Supplement: Supplementary file 3 [file Data_Sheet_3.pdf]

Participant involvement

Involvement of participants (diabetes specialist registrars) was discussed at various meeting of specialty bodies and especially at the specialist training forum called "Young diabetologist and endocrinologist forum" (more info about this forum at https://www.youngdiabetologists.org.uk/) with feedback received from committee members on the design and conduct of the study as well outcome measures and recruitment methodology for including the participants.

This was also discussed in deanery diabetes specialist registrars training meetings.

The plans for results dissemination of this study were also discussed and it was agreed that manuscript should be send for publication in an open access journal .
